# Oxygen Availability in Respiratory Muscles During Exercise in Children Following Fontan Operation

**DOI:** 10.3389/fped.2019.00096

**Published:** 2019-03-26

**Authors:** Fabian Stöcker, Rhoia Neidenbach, Celina Fritz, Renate M. Oberhoffer, Peter Ewert, Alfred Hager, Nicole Nagdyman

**Affiliations:** ^1^Department for Sport and Health Sciences, Teaching and Educational Center, Technical University Munich, Munich, Germany; ^2^Department of Pediatric Cardiology and Congenital Heart Defects, German Heart Centre, Munich, Germany; ^3^Department for Sport and Health Sciences, Chair of Preventive Pediatrics, Technical University Munich, Munich, Germany

**Keywords:** muscle oxygenation, Fontan, CHD, pediatrics, NIRS, respiratory muscles

## Abstract

**Introduction:** As survival of previously considered as lethal congenital heart disease forms is the case in our days, issues regarding quality of life including sport and daily activities emerge. In patients with Fontan circulation, there is no pump to propel blood into the pulmonary arteries since the systemic veins are directly connected to the pulmonary arteries. The complex hemodynamics of Fontan circulation include atrial function, peripheral muscle pump, integrity of the atrioventricular valve, absence of restrictive, or obstructive pulmonary lung function. Therefore, thoracic mechanics are of particular importance within the complex hemodynamics of Fontan circulation.

**Methods:** To understand the physiology of respiratory muscles, the aim of this study was to examine the matching of auxiliary respiratory muscle oxygen delivery and utilization during incremental exercise in young male Fontan patients (*n* = 22, age = 12.04 ± 2.51) and healthy Controls (*n* = 10, age = 14.90 ± 2.23). All subjects underwent a cardiopulmonary exercise test (CPET) to exhaustion whereas respiratory muscle oxygenation was measured non-invasively using a near-infrared spectrometer (NIRS).

**Results:** CPET revealed significantly lower peak power output, oxygen uptake and breath activity in Fontan patients. The onset of respiratory muscle deoxygenation was significantly earlier. The matching of local muscle perfusion to oxygen demand was significantly worse in Fontans between 50 and 90% $\dot{\text{V}}{\text{O}}_{2\text{peak}}$.

**Findings:** The results indicate that (a) there is high strain on respiratory muscles during incremental cycling exercise and (b) auxiliary respiratory muscles are worse perfused in patients who underwent a Fontan procedure compared to healthy Controls. This might be indicative of a more general skeletal muscle strain and worse perfusion in Fontan patients rather than a localized-limited to thoracic muscles phenomenon.

## Introduction

Functional univentricular congenital heart defects (CHD) entails different morphological diagnoses, the most common are hypoplastic left heart syndrome, tricuspid atresia, and double inlet left ventricle (1). In unpalliated univentricular CHD, cyanosis occurs because of mixing of unsaturated and saturated blood in the heart. The univentricular heart is exposed to volume overload as it drains both systemic and pulmonary venous return at the same time (2).

Palliation is achieved with the Fontan circulation (2). General principle is that the systemic venous return bypasses the subpulmonary ventricle with a separation of the systemic and pulmonary circulation and reduction of ventricular volume overload. Contemporary modifications of surgical techniques have significantly improved survival (2). A growing number of children and adults with congenital heart defects are living with a Fontan circulation with their limitations of chronic elevation of central venous pressure and restricted ventricular preload (3).

Patients with Fontan circulation are limited during exercise with a decreased peak VO_2_ compared to healthy controls (4). Healthy individuals increase their pulmonary blood flow during exercise by a reduction in pulmonary vascular resistance due to vasodilation and recruitment of segments and increased right ventricular work consisting of flow acceleration coupled with increased systolic pressures (5). In the Fontan patients, no pump ventricle exists to increase and accelerate pulmonary blood flow. Beyond this, pulmonary vascular reactivity and recruitment of vessels are limited or even absent (6). That leads to a restricted ability to boost cardiac output during exercise (6). The leading force of pulmonary blood flow in the absence of a pump ventricle is namely the negative pressure of the systemic atrium during ventricular systole and atrial volume increase due to the apical descent of a non-insufficient atrioventricular valve. Therefore, this physiology is even more dependent on the work of breathing to generate cardiac output (7). Active muscle contraction including the diaphragm increases the thoracic dimensions during inspiration generating a negative pressure and thus systemic blood flow into the thorax and of a passive decrease in thoracic dimensions based on the elastic recoil of lung itself. MRI studies estimated that ~30% of resting cardiac output is “respiratory dependent” while this dependency relies on the “thoracic pump” to increase cardiac out during exercise (8).

While understanding the hemodynamic differences between healthy individuals and Fontan patients, we know little about the peripheral muscle oxygenation in patients with univentricular circulation and the possible impact of an impaired ability to increase the oxygen supply adequately to the exercising muscles.

Few studies assessed the exercise capacity and respiratory muscle oxygenation in children with congenital heart diseases or studied the training effects on peripheral skeletal muscle oxygenation in children with congenital heart disease (9, 10). Till now, no study exists, which examined the respiratory muscle deoxygenation exclusively in children with Fontan circulation.

The aim of the study was to spot on the potential differences of respiratory muscle deoxygenation in children with Fontan circulation compared to healthy children during cardiopulmonary exercise testing (CPET) with near-infrared spectroscopy (NIRS).

## Materials and Methods

### Patients

From April 2014 to December 2016, 22 male patients (12.04 ± 2.51 year, range 12–18 year) with Fontan circulation (see Table 1) participated this study. They underwent a preliminary medical screening. From those 30 patients, 8 patients had to be excluded after the preliminary screening.

**Table 1:** Characteristics of fontans and controls.

	**Fontans (*n* = 22)**	**Controls (*n* = 10)**	***p*-value**
Age (y)	12.04 ± 2.51	14.90 ± 2.23	0.004
Body mass (kg)	39.68 ± 13.38	52.80 ± 11.08	0.011
Height (cm)	149.77 ± 14.83	167.00 ± 12.78	0.003
BMI (kg*m^−2^)	17.18 ± 2.42	18.68 ± 1.59	0.087
Skinfold thickness (cm)	6.50 ± 1.99	5.46 ± 1.80	0.173

*BMI, Body Mass Index; data in mean ± standard deviation*.

Underlying heart defects were hypoplastic left heart syndrome (*n* = 9), double outlet right ventricle (DORV) with transposition of the great arteries (TGA) and left ventricle outflow obstruction (*n* = 1), DORV with atrioventricular discordance and pulmonary stenosis (*n* = 1), DORV with hypoplastic left ventricle (*n* = 1), TGA with hypoplastic right heart and pulmonary stenosis (*n* = 1), double inlet left ventricle (*n* = 4), tricuspid atresia (*n* = 2), mitral atresia (*n* = 2), and pulmonary atresia with intact ventricular septum and coronary fistula (*n* = 1).

Fontan patients had at least two surgical procedures (mean 3.36, range 2–5). Median age at the Fontan procedure was 21.5 months (range 17–54 months). Median time interval from the Fontan procedure to the study was 9.12 years (range 6.62–14.51 years).

Two Fontan patients had a pacemaker due to bradycardia caused by sinus node dysfunction. Hemoglobin values were in a normal range in all Fontan patients (15.32 ± 1.25 gdl).There were no skeletal abnormalities as scoliosis in the Fontan patients.

All Fontan patients were in NYHA class II (*n* = 22). All Fontans participated to regular school sport activity, no one was excluded.

Transthoracic echocardiography revealed normal single ventricular function in 19 Fontan patients, while it was reduced in three patients. Four patients demonstrated moderate atrioventricular valve regurgitation, while the rest was mild or less. All patients were under oral anticoagulation and no thrombus could be detected by echocardiogram. ACE inhibitors were given in four, diuretics in three, digoxin and beta blockers in two patients each. None had pulmonary vasoactive medication.

Patients were in stable condition allowing exhaustive exercise.

The Controls consisted of 10 male healthy children (14.90 ± 2.23 year, range 10–17 year, Table 1) who underwent a routine check-up for participating in a sports club programme (Table 1). Each healthy subject did at least 2 h of physical exercise per week at school. Fontan patients and Controls did not suffer from other relevant diseases that might lead to an impaired muscle performance. None of the subjects were in any family relationship. Anthropometric data of Fontan patients and Controls is listed in Table 1.

The study conforms to the principles outlined in the Declaration of Helsinki 1975 and was approved by the Institutional Review Board of the Technical University of Munich (project number 52/14). Written informed consent was obtained from all study participants and parents.

### Cardiopulmonary Exercise Test (CPET)

All subjects underwent a CPET with a cycle ergometer (Corival, Lode, Groningen, The Netherlands) in upright position or in patients with a rate responsive pacer with a treadmill (CareFusion, LE 200 CE) according to international guidelines (11), with one of the authors present at all times.

After a 3 min baseline measurement at rest and further 3 min of unloaded cycling as warm-up, load was increased ramp-wise with 5, 10, 15, 20, or 30 W/min depending on the expected individual physical capacity estimated by the investigator. The aim was to reach cycle duration of 8–12 min after warm up until exhaustion was reached. The end of the CPET was marked by symptom limitation and was followed by a 5 min recovery period with the first 3 min cycling with minimal load and another 2 min at rest.

The following criteria considered the tests to be of maximal effort: a respiratory exchange ratio >1.0; exhaustion of the patient with an inability to maintain a cycle pedaling rate of 60·min^−1^.

Respiratory gas-exchanges were measured by a breath-by-breath analysis using a metabolic chart (Vyasis Healthcare Vmax Encore 29, Hochberg, Germany). The device was calibrated prior to each test according to manufacturer's recommendations.

Blood pressure was measured by an automated, ECG-triggered acoustic device (SunTech Medical Inc.: Tango M2, Morrisville, NC, USA) every 2 min during examination time. Heart rate and rhythm was monitored with a continuous ECG during the entire examination. Oxygen saturation (SpO_2_) was monitored in the Fontans continuously using a forehead sensor.

The ventilatory threshold (VT) was determined from the gas exchange data using the V-slope method validated by Beaver and colleagues (12). VT is defined as the breakpoint of the linearity of the curve carbondioxide elimination ($\dot{\text{V}}$CO_2_) vs. oxygen uptake ($\dot{\text{V}}$O_2_). This point was identified by two independent experienced observers.

Peak oxygen uptake ($\dot{\text{V}}$O_2peak_) was defined as the highest moving average 30 s interval during the exercise period. Reference values (mL^*^kg^−1*^min^−1^) were calculated according to Cooper and Weiler-Ravell (13).

For adolescents 12 years and older, sex-specific reference values (mL/kg/min) were calculated:

$$\begin{array}{l} \text{Female}:\;\dot{\text{V}}{\text{O}}_{2\text{peak}}=(22.5•\text{height }(\text{cm})-1837.8)/\text{weight}\;(\text{kg})\\ \text{ Male}:\;\dot{\text{V}}{\text{O}}_{2\text{peak}}=(43.6•\text{height }(\text{cm})-4547.1)/\text{weight}\;(\text{kg}) \end{array}$$

For patients younger than 12 years of age, reference values (mL^*^kg^−1*^min^−1^) were calculated from pooled data from both sexes:

$$\dot{\text{V}}{\text{O}}_{2\text{peak}}=(37.1•\text{height }(\text{cm})-3770.6)/\text{weight}\;(\text{kg})$$

### Near-Infrared Spectroscopy (NIRS) Measurement

The principles behind the NIRS technique is described in detail elsewhere (14, 15). In brief, NIRS measurements are based on the relative tissue transparency for light in the near-infrared spectrum and on the O_2_-dependent absorption changes of oxygenated and deoxygenated hemoglobin and myoglobin (ΔO_2_Hb+Mb, ΔHHb+Mb) based on the Lambert-Beer-law. Data was measured continuously using a wireless, continuous-wave near-infrared spectrometer (PortaLite, Artinis B.V., Zetten, NL) with a sample rate of 10 Hz. A differential pathlength-factor of 4 was used, as this has been reported as appropriate factor for muscle measurements previously (15). Accordingly, all NIRS data are expressed as Δμmol·L^−1^ and adjusted to pre-exercise values that were obtained during the 3 min baseline measurement prior to the cardiopulmonary exercise test.

The NIRS device operates with NIR wavelengths of 760 and 850 nm, while the analyzed emitter-detector distance was 3.5 cm. This results in a penetration depth of ~1.75 cm (15).

The NIRS probe was placed on the right side of the upper body above the sixth intercostal space at the anterior axillary line over the serratus anterior muscle. This position has been used previously to monitor oxygenation of this muscle (8). This muscle was chosen because it serves as an auxiliary inspiratory muscle during the ventilation as a “rib elevator”.

We measured the skinfold thickness by a fat caliper (GPM, DKSH Inc., Zürich, CH) over the serratus anterior muscle for interpretation the quality of the gained NIRS data.

### Data Processing

First, NIRS and CPET data were time-aligned. Therefore, the data were converted to 10 s bins and processed with a 30 s moving average. As mentioned previously NIRS data was calculated by subtracting-individual baseline values to be expressed for example as ΔHHb. Furthermore, the NIRS data was normalized to the individual peak values, which had been reached in the trial, to be expressed as %HHbmax. This means that in all subjects %HHbmax started at 0% at baseline and reaches 100% at the point of maximal respiratory muscle desoxygenation. As an estimate for the adjustment of microvascular perfusion, $\Delta \text{HHb}/\Delta \dot{\text{V}}{\text{O}}_{2}$ ratio was calculated according to %HHbmax/%$\dot{\text{V}}$O_2peak_ for each time point during the test. This parameter has been used in several previous studies (16–19).

### Statistical Analysis

A repeated-measures-design ANOVA with one group-factor *Fontans* vs. *Controls* was used to evaluate the effect of *intensity* on NIRS data and CPET data. Data was analyzed at 20, 30, 40, 50, 60, 70, 80, 90, and 100% $\dot{\text{V}}$O_2peak_. Repeated contrasts were used to analyze possible interaction-effects of *intensity x Fontans* vs. *Controls*. All *post-hoc* tests were Bonferroni-Holm corrected. Power output at VT, $\dot{\text{V}}$O_2peak_, peak power output, skinfold thickness were compared among the two groups using independent *t*-tests. Additionally, we divided the Fontans into two subgroups of left and right single ventricular morphologies and compared VO_2peak_, peak power output and muscle deoxygenation among both morphologies. The level of statistical significance was set to *p* < 0.05.

## Results

### Fontans and Controls

The detailed characteristics of the patients and control group are given in Table 1.

### CPET

We obtained resting, submaximal, and maximal cardiopulmonary variables in all Fontans and Controls while submaximal variables were taken at the similar exercise intensity.

At rest, Fontans had similar heart rate, $\dot{\text{V}}$O_2_, $\dot{\text{V}}$CO_2_, $\dot{\text{V}}$E, and blood pressure compared to healthy Controls.

$\dot{\text{V}}$O_2peak_, peak power output, $\dot{\text{V}}$CO_2peak_, $\dot{\text{V}}$E_peak_, and HR_peak_ were significantly reduced in the Fontans compared to healthy Controls (*p* < 0.01).

At VT, all parameters showed significant lower values in Fontans compared to the Controls. The healthy Controls had larger absolute values and reached VT later than the Fontans.

All data for CPET in comparison are given in Table 2.

**Table 2:** Parameters of CPET in fontan patients and healthy controls measured at the ventilatory threshold and at peak level.

	**Fontan patients (*n* = 22)**	**Healthy controls (*n* = 10)**	***p*-value**
Workload (W·kg^−1^) max	3.18 ± 1.13	4.35 ± 0.24	0.003*
Workload (W·kg^−1^) at VT	1.50 ± 0.34	1.53 ± 0.63	0.875
$\dot{\text{V}}$O_2_ (ml·min^−1^·kg-^1^) peak	35.97± 6.84	44.76 ± 5.09	0.001*
% VO_2peak_ at VT	36.14 ± 16.71	62.94 ± 9.61	< 0.001*
$\dot{\text{V}}$O_2peak_ % predicted	73.61 ± 17.69	86.87 ± 12.61	0.044*
$\dot{\text{V}}$E_peak_ (L·min^−1^)	60.29 ± 25.19	74.70 ± 18.12	0.010*
$\dot{\text{V}}$E_peak_ (L·min^−1^ m^−2^)	38.99 ± 9.97	47.12 ± 8.58	0.024*
$\dot{\text{V}}$E (L·min^−1^) at VT	29.29 ± 10.86	26.05 ± 7.80	0.404
$\dot{\text{V}}$E/$\dot{V}$CO_2_ Slope	32.04 ± 3.31	27.25 ± 5.41	0.004*
HR (beats·min) peak	171.20 ± 21.20	187.66 ± 5.16	0.002*
HR (beats·min) at VT	120.10 ± 16.17	118.50 ± 20.95	0.816
S_P_O_2_ (%) peak	87.59 ± 5.19	n.d.	
S_P_O_2_ (%) at VT	88.55 ± 7.37	n.d.	
Respiratory rate peak	51.68 ± 8.31	48.95 ± 11.13	0.444
Respiratory rate at VT	34.86 ± 5.99	26.25 ± 9.64	0.004

*Data in mean ± standard deviation*.

*Asterisks indicate significant differences between Fontans and Healthy Controls*.

### Respiratory Muscle Oxygenation by NIRS

ΔHHb was significantly influenced by exercise intensity in both groups [*F*_(3.8, 120.3)_ = 91.96, *p* ≤ 0.001], whereas the onset of muscle deoxygenation occurred differently: in the Controls, ΔHHb remained stable up to 50% $\dot{\text{V}}$O_2peak_. Above this intensity, ΔHHb increased significantly (*p* ≤ 0.012) until exhaustion (see Figure 2). In the Fontans, ΔHHb evolved similar, but ΔHHb increased significantly already above 40% $\dot{\text{V}}$O_2peak_ (*p* ≤ 0.027). The temporal dissociation between the increase of ΔHHb in the Fontan and control group was supported by a significant interaction effect of *intensity x Fontans* vs. *Controls* [*F*_(3.8, 120.3)_ = 4.7, *p* = 0.002]. Those results were confirmed by *post-hoc* tests. Between 50 and 90% $\dot{\text{V}}$O_2peak_, ΔHHb values were significantly higher for the Fontans compared to the Controls (*p* ≤ 0.028).

**Figure 1:**
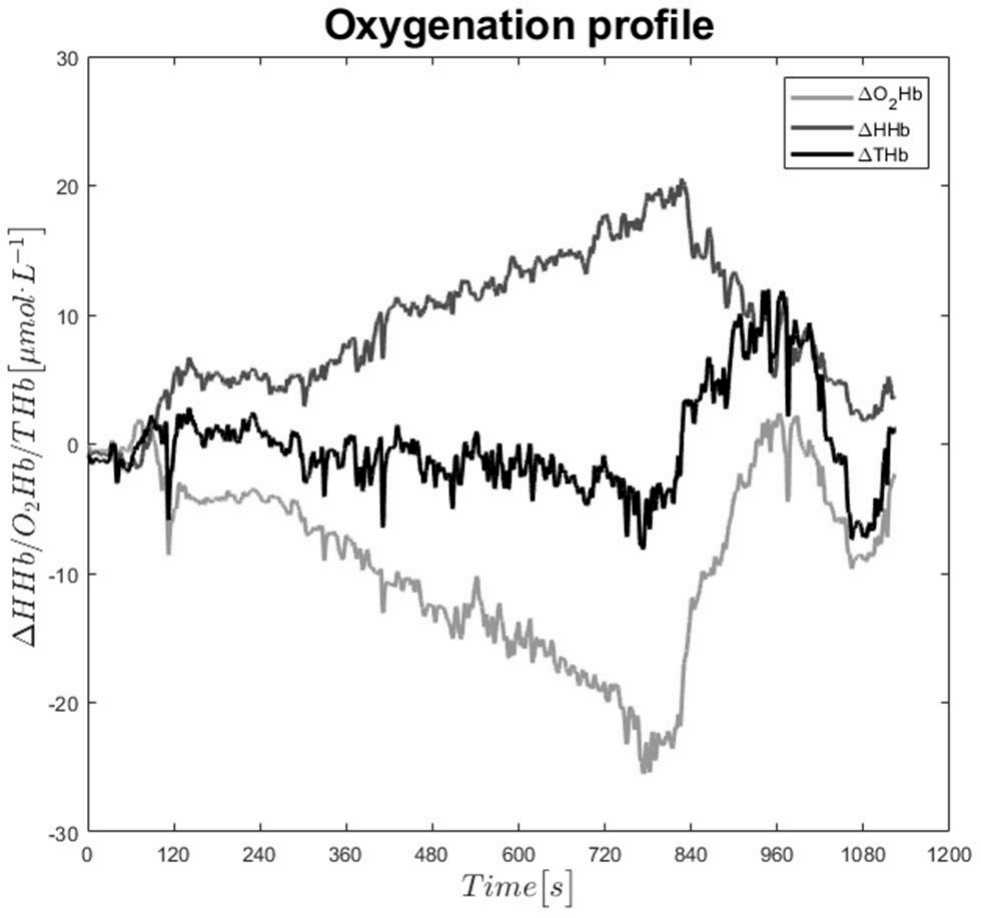
Exemplary time-course of oxygenated, deoxygenated, and total hemoglobin during the CPET.

**Figure 2:**
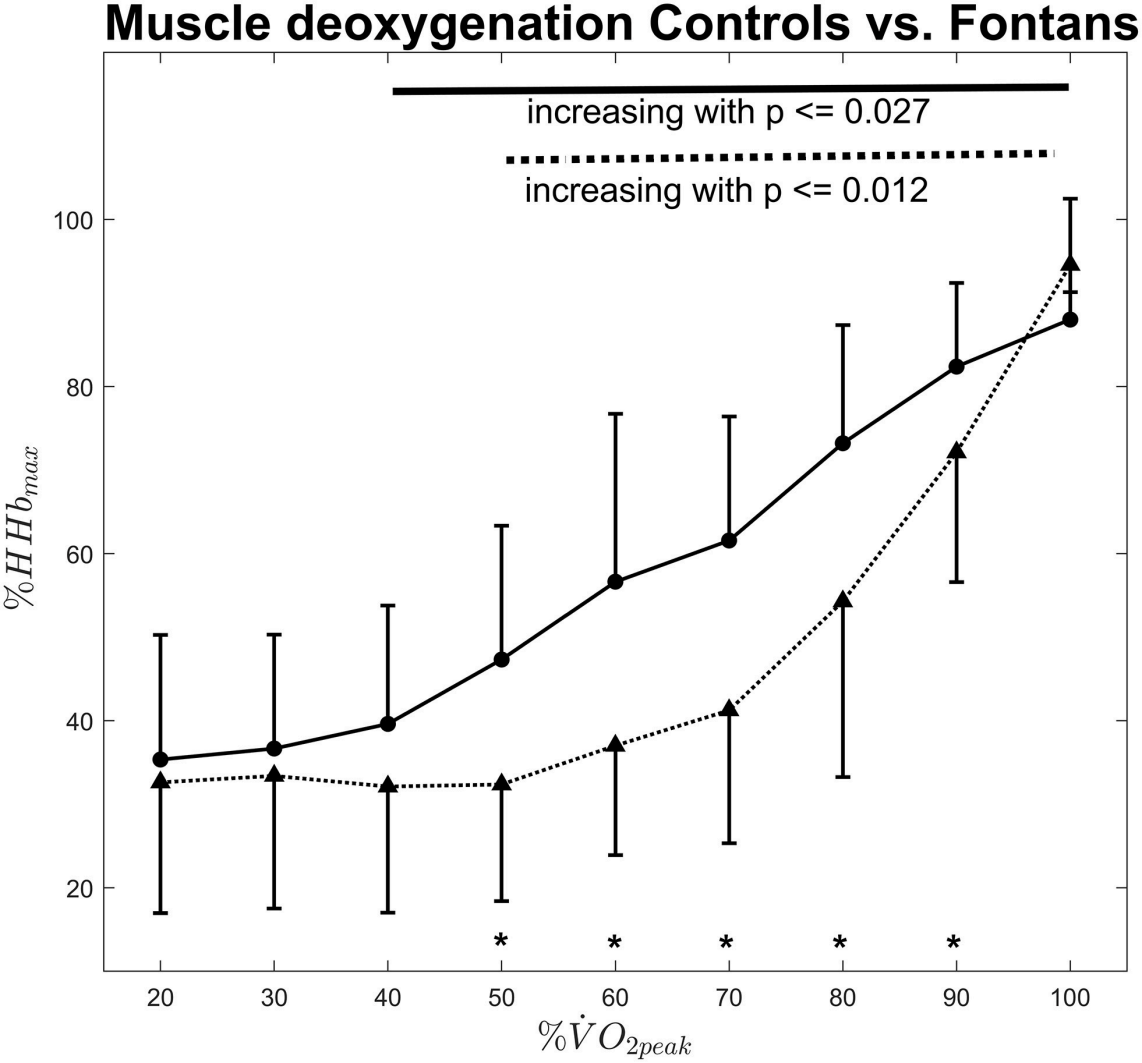
Normalized muscle deoxygenation vs. exercise intensity for Fontans (solid line) and Controls (dashed line). The solid and dashed lines in the upper section indicate significant differences between intensities among Fontans and Controls, respectively. Significant differences (*p* < 0.05) between Fontans and Controls within one exercise intensity are marked with asterisks.

**Figure 3:**
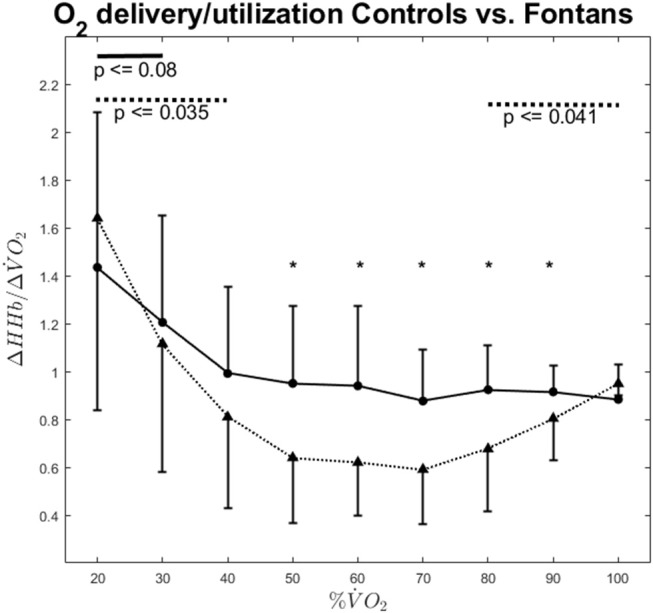
ΔHHb/Δ VO_2_ ratio vs. exercise intensity for Fontans (solid line) and Controls (dashed line). The solid and dashed lines in the upper section indicate significant differences between intensities among Fontans and Controls, respectively. Significant differences (*p* < 0.05) between Fontans and Controls within one exercise intensity are marked with asterisks.

Exercise intensity also had a significant influence on ΔHHb/$\Delta \dot{\text{V}}$O_2_ ratio [*F*_(2.3, 75.0)_ = 91.96, *p* ≤ 0.001]. In Fontans, ΔHHb/$\Delta \dot{\text{V}}$O_2_ ratio decreased significantly up to an exercise intensity of 40% $\dot{\text{V}}$O_2peak_ whereas no significant changes occurred at higher exercise intensities (see Figure 3). Contrary, ΔHHb/$\Delta \dot{\text{V}}$O_2_ ratio evolved “u-shaped” for the control group: After a significant decrease with increasing exercise intensity up to 50% $\dot{\text{V}}$O_2peak_ (*p* ≤ 0.035), ΔHHb/$\Delta \dot{\text{V}}$O_2_ ratio remained stable up to 70% $\dot{\text{V}}$O_2peak_ but increased thereafter (*p* ≤ 0.041). ΔHHb/$\Delta \dot{\text{V}}$O_2_ ratio did not differ among both groups at low (20–40% $\dot{\text{V}}$O_2peak_) and maximum exercise intensity. In between, the Fontan group showed significantly higher values compared to those of the control group (*p* ≤ 0.031).

**Figure 4:**
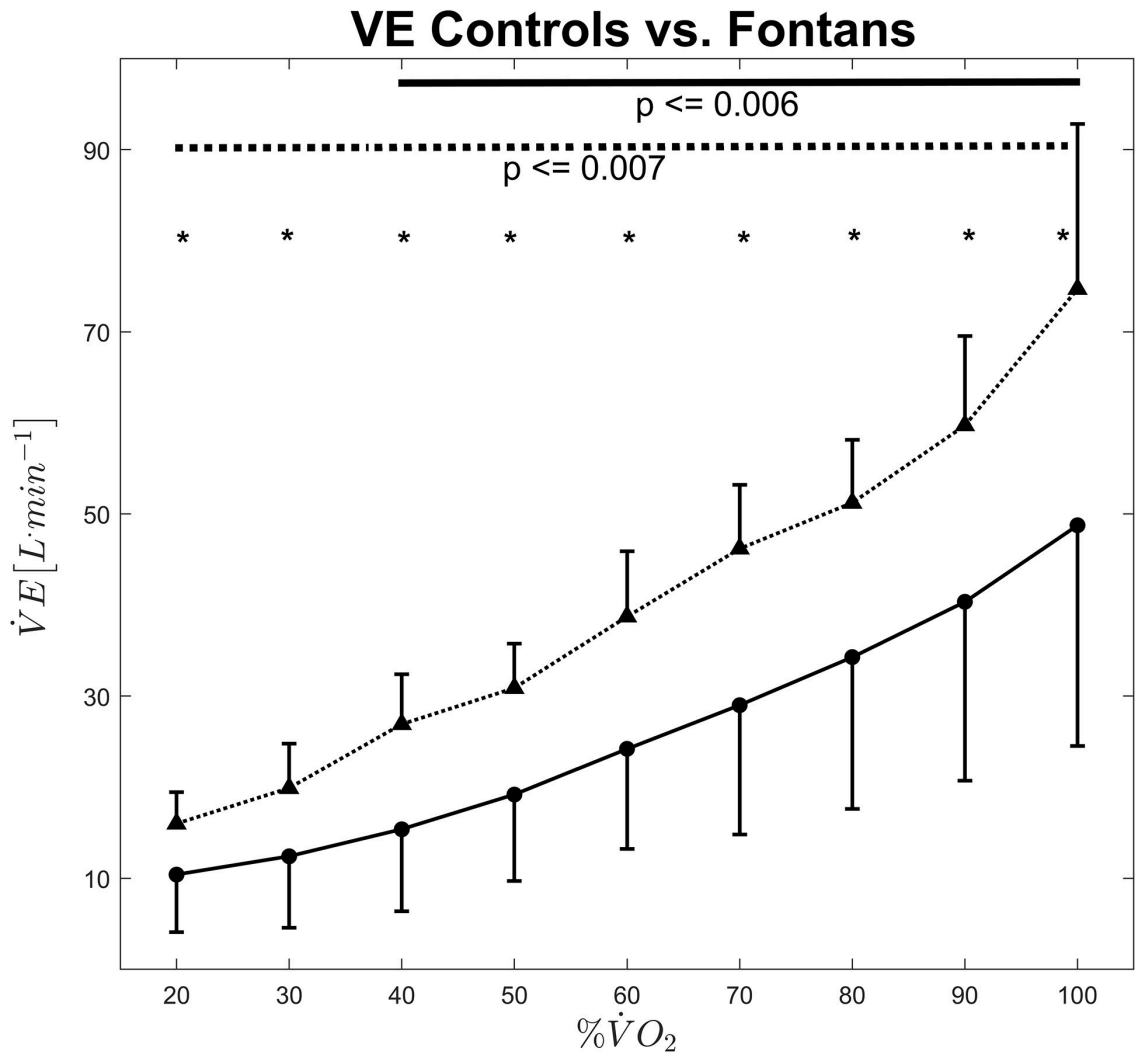
Ventilation vs. exercise intensity for Fontans (solid line) and Controls (dashed line). The solid and dashed lines in the upper section indicate significant differences between intensities among Fontans and Controls, respectively. Significant differences (*p* < 0.05) between Fontans and Controls within one exercise intensity are marked with asterisks.

Ventilation increased significantly with exercise intensity [*F*_(1.6, 52.5)_ = 127.1, *p* ≤ 0.001] whereas the stepwise increases were significant above 40% $\dot{\text{V}}$O_2peak_ (*p* ≤ 0.006) for the Fontans and right from the beginning (*p* ≤ 0.007) for the Controls (see Figure 4). While no interaction could be observed for *intensity x Fontans* vs. *Controls*, ventilation was greater in the control group at all tested intensities [ANOVA: *F*_(1.6, 52.5)_ = 4.7, *p* = 0.018; *post-hoc* tests: *p* ≤ 0.013]. We analyzed ventilation also relatively to body surface area (L^*^m^−2^). Body surface area was calculated according to Dubois (20). Relative ventilation also increased significantly above 40% $\dot{\text{V}}$O_2peak_ in Fontans (*p* ≤ 0.006) and right from the beginning in the control group (*p* ≤ 0.007). Again, no interaction could be shown but relative ventilation was significantly higher in seven out of the nine examined intensities (40–100% $\dot{\text{V}}$O_2peak_; *p* ≤ 0.031).

Peak power was significantly lower in Fontans compared to healthy Controls (3.18 ± 1.13 vs. 4.35 ± 0.24 W·kg^−1^, *p* = 0.003), just as $\dot{\text{V}}$O_2max_ (35.97 ± 6.84 vs. 44.76 ± 5.09 ml·min^−1^·kg^−1^, *p* = 0.001).

After dividing the Fontans into two subgroups of right and left single ventricular morphologies (RVs: 8 subjects, LVs 14 subjects), VO_2peak_ (34.67 ± 5.11 ml·min^−1^·kg^−1^ vs. 38.15 ± 9.04 ml·min^−1^·kg^−1^) and Peak Power were higher (98 ± 25.80 W vs. 141.88 ± 56.25 W) in patients with a LV morphology, while this difference was only significant for Peak Power (*p* = 0.029). No significant effect of the heart morphology could be found on muscle oxygenation during exercise.

Skinfold thickness at the NIRS probe position was 3.49 ± 1.36 mm. The interoptode distance that we used in the study was 3.5 cm which corresponds to an approximate penetration depth of 1.75 cm. Hence, the NIRS signal can be referred to muscle tissue in the intercostal space.

## Discussion

The results of our study clearly show that there is high aerobic demand during cycling exercise, not only on working muscles but also on respiratory muscles. This was indicated by an inflection point in the ΔHHb signal, representing the onset of muscle deoxygenation (Figure 1). This inflection point was significantly different in children with Fontan palliation compared to healthy controls (40 $\dot{\text{V}}$O_2peak_ vs. 50% $\dot{\text{V}}$O_2peak_). NIRS signal represents the dynamic balance of local oxygen extraction and blood flow (i.e., oxygen supply). Therefore, an increase of ΔHHb indicates a higher local oxygen extraction in relation to oxygen supply. Our data hence reveal that this dynamic balance alternates earlier in Fontans compared to Controls.

These results are similar compared to those of Moalla et al. (9), who investigated 12 patients with various congenital heart defects. By normalizing data to the maximum deoxygenation that was reached in the graded exercise test, we focused more on the relationship of muscle deoxygenation kinetics rather than oxygen saturation as an absolute value. Therefore, we could identify the different onsets of muscle deoxygenation in Fontans vs. healthy Controls. Moalla et al. found the onset of pronounced muscle deoxygenation to be concomitant to VT (21). The onset on muscle deoxygenation that was measured in this study therefore could indicate somehow the “local” VT for the serratus anterior muscle.

When deoxygenation is expressed relatively to oxygen uptake (ΔHHb/$\Delta \dot{\text{V}}$O_2_-ratio), the matching of oxygen delivery to utilization can be estimated (16, 17, 22–24). ΔHHb/$\Delta \dot{\text{V}}$O_2_-ratio was significantly lower in Controls between 50 and 90% $\dot{\text{V}}$O_2peak_, suggesting a significantly higher local oxygen provision. In brief, the idea behind this parameter is that ΔHHb, which represents the dynamic balance between local oxygen extraction and desoxy-Hb removal (i.e., blood flow) is assumed to increase when $\dot{\text{V}}$O_2_ rises if this can be (partially) referred to the muscle of interest. If local blood flow would rise correspondingly, no increase in ΔHHb would be visible. However, local oxygen extraction usually exceeds the increase in local blood flow. Consequently, the smaller ΔHHb at a given workload/$\dot{\text{V}}$O_2_ is, the higher should be the local muscle perfusion and, hence, oxygen provision. However, the concept of ΔHHb/$\Delta \dot{\text{V}}$O_2_ originally was used for the estimation of microvascular oxygen provision in working muscles (24). We are aware that the serratus anterior muscle does not account for the major amount of consumed oxygen during cycling exercise. Hence, ΔHHb/$\Delta \dot{\text{V}}$O_2_ as an estimate for microvascular perfusion in the serratus anterior muscle has to interpreted with caution. However, data show clearly that ventilation increases consistently with exercise intensity. Concomitantly to exercise intensity, thigh muscle work increases. Consequently, a drop in ΔHHb/$\Delta \dot{\text{V}}$O_2_ it is very likely due to an enhanced local muscle perfusion. The m. serratus anterior is an auxiliary respiratory muscle that is supposed to support respiration especially under increased respiratory activity (25). Therefore, we think that ΔHHb/$\Delta \dot{\text{V}}$O_2_-ratio can be applied as a measure for respiratory muscle oxygen provision in the m. serratus anterior.

The reasons for this earlier onset of muscle deoxygenation and impaired matching of oxygen delivery and utilization might be explained by the response of the heart rate during exercise, as chronotropic incompetence could potentially explain part of this findings with the lack of increasing the cardiac output and thereby increased ΔHHb and decreased ΔHHb/$\Delta \dot{\text{V}}$O_2_-ratio earlier on exercise compared to healthy controls. Beside this, pulmonary vascular reactivity might be impaired, pulmonary vessel recruitment limited or even absent compared to healthy Controls (3).

In both groups, the matching of oxygen delivery to utilization improved during the initial phase of the incremental exercise test. However, this improvement stopped above 30% $\dot{\text{V}}$O_2peak_ in Fontans and above 40% $\dot{\text{V}}$O_2peak_ in Controls, respectively. This was close to VT (Fontans: 36.14 ± 16.71% $\dot{\text{V}}$O_2peak_; Controls: 62.94 ± 9.61% $\dot{\text{V}}$O_2peak_). Previous research showed that local oxygen availability in working muscles is improved following exercise bouts above VT (18), presumably due to greater local vasodilation. In the current study, ΔHHb/$\Delta \dot{\text{V}}$O_2_-ratio does improve further at intensities above VT.

The reasons for the improved ΔHHb/$\Delta \dot{\text{V}}$O_2_-ratio at low exercise intensities are likely due to the presence of more vasoactive substances, causing a greater local vasodilation (17). Various endothelial mediated pathways contribute to local vasodilation (26–28). Especially shear stress is to mention as important trigger for vasoactive substance release at higher workloads (29). Endothelial shear stress occurs when perfusion increases. In respiratory muscles, this can happen when breathing activity increases in response to the onset of exercise and therefore could explain why the matching of oxygen delivery and utilization improves across the lower exercise intensities. However, the ΔHHb/$\Delta \dot{\text{V}}$O_2_-ratio of the serratus anterior muscle stopped improving at exercise intensities close to VT. It therefore shows an inverted shape compared to the ΔHHb/$\Delta \dot{\text{V}}$O_2_-ratio that was measured previously in in working muscles during cycling exercise (18). One could speculate that this is due to the increasing exercise induced vasodilation in the working muscles above VT. It therefore would represent the blood flow redistributions to body regions with higher metabolic demands. Above VT, lactate is considered to support local vasodilation (30, 31) which could be an explanation for the promotion of local blood supply above VT e.g., to thigh muscles compared to respiratory muscles.

Besides the differences in microvascular hemodynamics, Fontan patients showed lower exercise tolerance and $\dot{\text{V}}$O_2peak_, which is in accordance to previous literature (32). Ventilation was also lower in Fontan patients, even when it was expressed relatively to body surface. Former studies showed significantly reduced lung volume and weaker respiratory muscles (33). Beside a lack of exercise compared to healthy Controls, the number of thoracotomies has been suggested to account for this restricted lung patterns (34). The higher respiratory muscle activity in healthy subjects could have contributed to the better estimated microvascular perfusion by greater endothelial mediated vasodilation caused by higher shear stress on the one hand and by a greater muscle-pump-effect (28) on the other hand.

Of course, the muscular under-performance might be due to suboptimal hemodynamics like failure to increase cardiac output, reduced increase in heart rate and indicative of a general muscular under-performance. If we would postulate this, the clinical implication of respiratory muscle oxygenation study would be of less clinical significance. Clinically, all our Fontans were in a good condition classified as NYHA II. However, training of respiratory muscles in the Fontan patients might improve the thoracic cavity mechanics under suboptimal hemodynamics, their pulmonary capacity and potentially also their microvascular perfusion. This could have a substantial effect in the management of patients with Fontan circulation. The next step would be to study the training effects on respiratory muscles in Fontans to figure out if there might be an improvement in microvascular perfusion.

Following a Fontan procedure, the heart's morphology results in an univentricular heart, either with the original left ventricle or the original right ventricle as the systemic ventricle. Because the left ventricle appears to be more powerful, the resulting heart morphology is of great importance. However, although VO_2peak_ and peak power output was significantly greater in Fontans with a left ventricle as the systemic ventricle, no differences could be observed regarding the muscle deoxygenation.

### Study Limitations

The control group was significantly older than the Fontans which also indicates an unbalanced distribution of sexual maturity in both groups. This was due to dropouts, which substantially distorted the original distribution. We do not think that this bias affects the outcome too much for two reasons. First, the subjects age range of both groups overlaps significantly, indicating that both groups are recruited out of the same age-group. Of course, this does not solve the issue that the age distribution is unbalanced. Second, we addressed this issue by normalizing all relevant measures. Furthermore, we correlated relative respiratory muscle deoxygenation (%HHb_max_) with age at 50% $\dot{\text{V}}$O_2peak_. This intensity evolved as the crucial intensity, where the onset of muscle deoxygenation has already happened in the Fontan group, but not in the control group. However, age was not significantly correlated with relative muscle deoxygenation (*r* = −0.14 for Fontans, *r* = −0.37 for Controls). Therefore, we assume that results were not influenced by the slightly different age distribution in both groups. In general, NIRS-derived signals are influenced by subcutaneous adipose tissue thickness (ATT) (35). To measure muscle oxygenation, the penetration depth, which is roughly half of the optode distance (15, 35), has to substantially exceed ATT. We used an inter-optode distance of 3.5 cm in this study, while ATT was 6.50 mm in Fontans and 5.46 mm in healthy Controls, respectively. Therefore, ATT should not impair NIRS-derived measures in this study.

Compared to adults, young children are mainly diaphragmatic breathers, meaning that when they face increased breathing work, they depend also to diaphragmatic contraction. The diaphragmatic function as major contributor to ventilation mechanics was not studied in our study. With the serratus anterior muscle, we analyzed an accessory muscle, probably not so much utilized in Fontan patients but comes to the fore when patients follow a respiratory muscle training.

A main issue is whether the oxygen saturation changes in the auxiliary respiratory muscles is indicative for a local response or it is indicative of a very similar or identical response of other muscles involved during the treadmill test. We did not compare the performance of respiratory muscles and skeletal muscles by using the NIRS technique on the same subjects in both cases and controls.

## Conclusion

This study shows that that Fontan patients limitation in exercise capacity is not only due to limited oxygen provision in primary working muscles e.g., in arms and legs but also to auxiliary muscles like respiratory muscles. Therefore, both peripheral working muscles and respiratory muscles should be trained in order to improve local vascular function. Furthermore, respiratory muscle training could be beneficial especially for Fontan patients because in could support the passive blood flow for the pulmonary circulation.

## Data Availability

The datasets generated for this study are available on request to the corresponding author.

## Author Contributions

FS supervised the NIRS measurements, did the data processing and was mainly responsible for drafting the manuscript. RN and CF conducted the CPETs. RO and PE provided resources for research. AH was responsible for the general experimental procedures and was revised the drafting process. NN was the project leader and coordinated the study and co-wrote the manuscript. All authors take responsibility for all aspects of the reliability and freedom from bias of the data presented and their discussed interpretation.

### Conflict of Interest Statement

The authors declare that the research was conducted in the absence of any commercial or financial relationships that could be construed as a potential conflict of interest.

## References

[1] Schwedler G Lindinger A Lange PE Sax U Olchvary J Peters B. Frequency and spectrum of congenital heart defects among live births in Germany: a study of the competence network for congenital heart defects. Clin Res Cardiol. (2011) 100:1111–7. 10.1007/s00392-011-0355-7 21909849

[2] Ven van der JPG van den Bosch E Bogers AJCCJ Helbing WA State of the art of the Fontan strategy for treatment of univentricular heart disease. F1000Res. (2018) 7:F1000 10.12688/f1000research.13792.1 PMC6024235 30002816

[3] Jolley M Colan SD Rhodes J DiNardo J. Fontan physiology revisited. Anesth Analg. (2015) 121:172–82. 10.1213/ANE.0000000000000717 26086514

[4] Gewillig M Goldberg DJ. Failure of the fontan circulation. Heart Fail Clin. (2014) 10:105–16. 10.1016/j.hfc.2013.09.010 24275298

[5] Van de Bruaene A La Gerche A Claessen G De Meester P Devroe S Gillijns H. Sildenafil improves exercise hemodynamics in Fontan patients. Circ Cardiovasc Imaging. (2014) 7:265–73. 10.1161/CIRCIMAGING.113.001243 24478333

[6] Gewillig M Brown SC. The Fontan circulation after 45 years: update in physiology. Heart. (2016) 102:1081–6. 10.1136/heartjnl-2015-307467 27220691 PMC4941188

[7] Hsia TY Khambadkone S Redington AN Migliavacca F Deanfield JE de MR. Effects of respiration and gravity on infradiaphragmatic venous flow in normal and Fontan patients. Circulation. (2000) 102:III148–53. 10.1161/01.CIR.102.suppl_3.III-148 11082378

[8] Fogel MA Weinberg PM Hoydu A Hubbard A Rychik J Jacobs M. The nature of flow in the systemic venous pathway measured by magnetic resonance blood tagging in patients having the Fontan operation. J Thorac Cardiovasc Surg. (1997) 114:1032–41. 10.1016/S0022-5223(97)70017-5 9434698

[9] Moalla W Dupont G Temfemo A Maingourd Y Weston M Ahmaidi S. Assessment of exercise capacity and respiratory muscle oxygenation in healthy children and children with congenital heart diseases. Appl Physiol Nutr Metabol. (2008) 33:434–40. 10.1139/H07-196 18461095

[10] Moalla W Elloumi M Chamari K Dupont G Maingourd Y Tabka Z. Training effects on peripheral muscle oxygenation and performance in children with congenital heart diseases. Appl Physiol Nutr Metabol. (2012) 37:621–30. 10.1139/h2012-036 22554184

[11] Gibbons RJ Balady GJ Bricker JT Chaitman BR Fletcher GF Froelicher VF. ACC/AHA 2002 guideline update for exercise testing: summary article: a report of the American College of Cardiology/American Heart Association Task Force on Practice Guidelines (Committee to Update the 1997 Exercise Testing Guidelines). Circulation. (2002) 106:1883–92. 10.1161/01.CIR.0000034670.06526.15 12356646

[12] Beaver W Wasserman K Whipp B. A new method for detecting anaerobic threshold by gas exchange. J Appl Physiol. (1986) 60:2020–7. 3087938 10.1152/jappl.1986.60.6.2020

[13] Cooper DM Weiler-Ravell D. Gas exchange response to exercise in children. Am Rev Respir Dis. (1984) 129:S47–8. 10.1164/arrd.1984.129.2P2.S47 6696341

[14] Ferrari M Binzoni T Quaresima V. Oxidative metabolism in muscle. Philos Trans R Soc London Series B: Biol Sci. 352:677–83. 10.1098/rstb.1997.0049 9232855 PMC1691965

[15] Ferrari M Mottola L Quaresima V. Principles, techniques, and limitations of near infrared spectroscopy. Can J Appl Physiol. (2004) 29:463–87. 10.1139/h04-031 15328595

[16] DeLorey DS Kowalchuk JM Paterson DH Effect of age on O_2_ uptake kinetics and the adaptation of muscle deoxygenation at the onset of moderate-intensity cycling exercise. J Appl Physiol. (2004) 97:165–72. 10.1152/japplphysiol.01179.2003 15003999

[17] Murias JM Kowalchuk JM Paterson DH. Speeding of VO_2_ kinetics with endurance training in old and young men is associated with improved matching of local O_2_ delivery to muscle O_2_ utilization. J Appl Physiol. (2010) 108:913–22. 10.1152/japplphysiol.01355.2009 20150562 PMC2853203

[18] Stöcker F Oldershausen VC Paternoster F Schulz T Oberhoffer R End-exercise ΔHHb/ΔVO 2and post-exercise local oxygen availability in relation to exercise intensity. Clin Physiol Funct Imaging. (2015) 37:384–393. 10.1111/cpf.12314 26576503

[19] Stöcker F Von Oldershausen C Paternoster FK Schulz T Oberhoffer R. Does postexercise modelled capillary blood flow accurately reflect cardiovascular effects by different exercise intensities? Clin Physiol Funct Imaging. (2017) 38:431–8. 10.1111/cpf.12434 28444930

[20] Bois D Du Bois EF Du. A formula to estimate the approximate surface area if height and weight be known. 1916. Nutrition. (1989) 5:303–11. 2520314

[21] Moalla W Dupont G Berthoin S Ahmaidi S. Respiratory muscle deoxygenation and ventilatory threshold assessments using near infrared spectroscopy in children. Int J Sports Med. (2005) 26:576–82. 10.1055/s-2004-830332 16195992

[22] Spencer MD Murias JM Grey TM Paterson DH Regulation of VO_2_ kinetics by O_2_ delivery: insights from acute hypoxia and heavy-intensity priming exercise in young men. J Appl Physiol. (2012) 112:1023–32. 10.1152/japplphysiol.01215.2011 22194321

[23] Poole DC Richardson RS. Determinants of oxygen uptake. Implications for exercise testing. Sports Med. (1997) 24:308–20. 9368277 10.2165/00007256-199724050-00003

[24] DeLorey DS Kowalchuk JM Paterson DH. Relationship between pulmonary O2 uptake kinetics and muscle deoxygenation during moderate-intensity exercise. J Appl Physiol. (2003) 95:113–20. 10.1152/japplphysiol.00956.2002 12679363

[25] Flaminiano LE Celli BR. Respiratory muscle testing. Clin Chest Med. (2001) 22:661–77. 10.1164/rccm.166.4.518 11787658

[26] Joyner MJ Casey DP. Regulation of increased blood flow (Hyperemia) to muscles during exercise: a hierarchy of competing physiological needs. Physiol. Rev. 95:549–601. 10.1152/physrev.00035.2013 25834232 PMC4551211

[27] Haram P Adams V Kemi O Brubakk AO Hambrecht R Ellingsen O. Time-course of endothelial adaptation following acute and regular exercise. Eur J Cardiovasc Prevent Rehabil. (2006) 13:585–91. 10.1097/01.hjr.0000198920.57685.76 16874149

[28] Tschakovsky ME. Immediate exercise hyperemia: contributions of the muscle pump vs. rapid vasodilation. J Appl Physiol. (2004) 97:739–47. 10.1152/japplphysiol.00185.2004 15247202

[29] Tinken TM Thijssen DH Hopkins N Dawson EA Cable TN Green DJ. Shear stress mediates endothelial adaptations to exercise training in humans. Hypertension. 55:312–8. 10.1161/HYPERTENSIONAHA.109.146282 20048193

[30] Gerbino A Ward S Whipp B. Effects of prior exercise on pulmonary gas-exchange kinetics during high-intensity exercise in humans. J Appl Physiol. (1996) 80:99–107. 8847338 10.1152/jappl.1996.80.1.99

[31] Chen Y Wolin M Messina E. Evidence for cGMP mediation of skeletal muscle arteriolar dilation to lactate. J Appl Physiol. (1996) 81:349–54. 8828684 10.1152/jappl.1996.81.1.349

[32] Brassard P Bédard É Jobin J Rodés-Cabau J Poirier P. Exercise capacity and impact of exercise training in patients after a Fontan procedure: a review. Can J Cardiol. (2006) 22:489–95. 10.1016/S0828-282X(06)70266-5 16685313 PMC2560550

[33] Turquetto A Canêo L Agostinho D Oliveira P Lopes M Trevizan P. Impaired pulmonary function is an additional potential mechanism for the reduction of functional capacity in clinically stable fontan patients. Pediatric Cardiol. (2017) 38:981–90. 10.1007/s00246-017-1606-9 28500413

[34] Müller J Ewert P Hager A. Number of thoracotomies predicts impairment in lung function and exercise capacity in patients with congenital heart disease. J Cardiol. (2018) 71:88–92. 10.1016/j.jjcc.2017.05.005 28687271

[35] van Beekvelt M Borghuis M van Engelen B Wevers R Colier WN. Adipose tissue thickness affects *in vivo* quantitative near-IR spectroscopy in human skeletal muscle. Clin Sci. (2001) 101:21–28. 10.1042/CS20000247 11410110

